# Case Report of Ultrasonographic and Computed Tomography Findings of Presumed Traumatic Ocular Globe and Lens Rupture in a Cat

**DOI:** 10.1155/crve/2106559

**Published:** 2025-08-07

**Authors:** Julius Klever, Clara Koch, Maria-Christine Fischer

**Affiliations:** ^1^Centre of Veterinary Clinical Medicine, Ludwig-Maximilians-University, Munich, Germany; ^2^Small Animal Specialist Hospital, Sydney, Australia; ^3^Queen Mother Hospital for Animals, Royal Veterinary College, London, UK

**Keywords:** computed tomography, globe rupture, lens rupture, ophthalmology, orbital trauma, ultrasound

## Abstract

A 5-year-old female spayed domestic short hair cat was presented 2 weeks after a history of unknown trauma. The examination of the head revealed facial asymmetry. Vision tests, including menace responses, were negative. Pupillary light reflexes and dazzle reflexes were negative in both eyes. The posterior segment of the eye could not be visualised due to miosis, hyphaema and fibrin. Ultrasound and computed tomography were performed to assess the eyes in detail. Based on computed tomography and ultrasound, a right ocular globe rupture, left ocular lens luxation and rupture and bilateral vitreal haemorrhage were diagnosed. This case report demonstrates the important role of ultrasound and computed tomography to assess orbital trauma in patients with limited visibility of the posterior segment of the eye due to hyphaema and describes imaging findings of globe and lens rupture.

## 1. Introduction

Craniofacial injuries are frequently seen in feline patients after trauma. Reasons are often car accidents or falling from great heights [1]. Blunt trauma may lead to globe proptosis and/or globe rupture. Globe ruptures frequently occur in the cornea or sclera, but can also affect the posterior wall of the globe. Cats are more predisposed to globe ruptures than large animals because of the anatomical open conformation of the orbit and the small body size [2]. Diagnosis of globe rupture can be challenging, especially if located posteriorly, because it cannot be visualised in the ophthalmologic examination; therefore, diagnostic imaging is usually performed. Not in every case can the site of rupture be clearly identified, but compatible alterations on ocular ultrasound such as irregular scleral contour, decreased reflectivity of the sclera and intraocular echoic or hyperechoic material can be seen [3]. Uveitis, hyphaema, vitreal haemorrhage, retinal detachment, iris bombe, traumatic cataract, endophthalmitis and phthisis bulbi may occur secondary to globe rupture [4].

Moderate or severe blunt trauma to the globe can lead to lens damage by coup and contrecoup injuries or equatorial expansion. Ocular emergencies involving the lens are not common but need to be recognised quickly, as a disruption of the lens capsule may result in undesirable sequelae [2]. Lens capsule rupture leads to phacoclastic uveitis caused by lens protein [5]. While defects of the capsule may not be seen, fibrinous or inflammatory cellular material is suggestive of capsular disruption. Ultrasound of lens capsule rupture in diabetic cataract has been described [6]. Unfortunately, medical therapy of phacoclastic uveitis with high doses of topical prednisone and topical atropine is often insufficient, and phacoemulsification may be necessary [7].

As head trauma can be associated with life-threatening conditions, the patient should be assessed for fractures and neurological disorders prior to the assessment of ocular injuries. In the case of orbital trauma, an ophthalmologic examination should be performed to evaluate ocular injuries. Further information on the cause of vision loss and prognosis for its return may play an essential role in decision-making due to the impact of vision on the quality of life. This is especially important if both globes are affected and the animal is blind at presentation. Cats with orbital trauma often present with hyphaema, which can be an indication for cross-sectional diagnostic imaging of the eye due to limited visibility of posterior ocular anatomical structures [2]. Both computed tomography (CT) and ultrasound have been shown to be valuable in the diagnosis of ocular disease [8, 9]. As globe rupture appears very painful and ultrasound may increase the risk of extrusion, a noncontact imaging examination technique may be preferable. Three-dimensional cross-sectional imaging is especially helpful to determine the extent of injuries. CT is a useful tool to evaluate craniofacial injuries, including orbital fractures, temporomandibular joint injuries, skull fractures and sometimes brain haemorrhage [1]. In feline patients with a reduced level of consciousness, it might be more appropriate to avoid sedation and perform CT using the VetMouseTrap™, a device that allows CT of awake cats [10].

## 2. Case Presentation

### 2.1. Signalement and History

A 5-year-old female spayed domestic short hair cat was presented 2 weeks after unknown trauma. The cat was reported to be healthy before the trauma. Prior to presentation at the university teaching hospital, primary care was supplied by the referring veterinarian. Initial bilateral epistaxis, bilateral hyphaema and a mandibular symphysial separation were reported. Surgical fixation of the symphysial separation with a cerclage wire was performed, and an oesophagostomy tube was placed by the referring veterinarian 10 days prior to presentation. The cat was referred for CT after severe malocclusion did not resolve following surgery.

### 2.2. Clinical Examination

At presentation, the patient appeared depressed. Vital signs were within normal limits (heart rate: 180/min; respiratory rate: 24/min; body temperature: 38.0°C; body weight: 4.6 kg; body condition score: 5/9), and mucous membranes were pink and moist. No palatal soft tissue injury was noted. Limited range of motion of the jaw and deviation of the mandibles to the left side were noted. Teeth were intact, and mandibular and cervical lymph nodes were normal. Thoracic auscultation was normal. No heart murmur or abnormal heart sounds were detected. Abdominal palpation was unremarkable. Soft tissue swelling and pus were evident at the oesophagostomy site; therefore, the feeding tube was removed immediately. An intravenous catheter was placed, and blood for complete blood count (CBC) and blood chemistry profile was collected. Results revealed lymphocytosis (WBC 21.2 × 10^3^/mm^3^) most likely related to the purulent oesophagostomy site.

### 2.3. Ophthalmologic Examination

**Figure 1:**
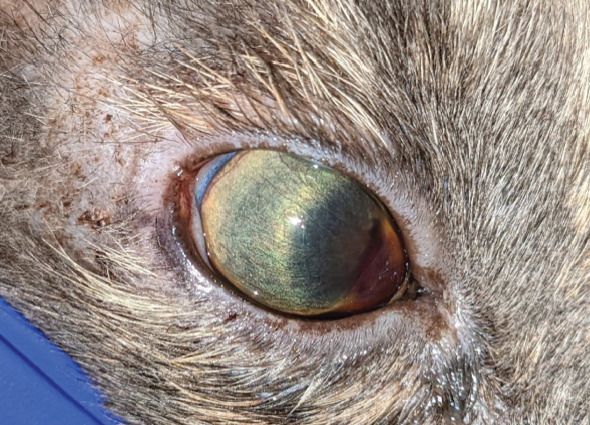
Right eye. Note the shallow anterior chamber with anterior bulging of the iris, the medially displaced miotic pupil and the hyphaema and fibrin.

**Figure 2:**
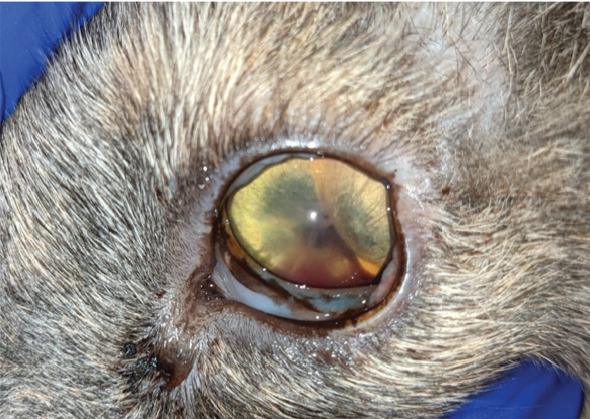
Left eye. Note the iris bombe resulting in anterior bulging of the iris and the uveitis with fibrin in hyphaema.

The examination of the head revealed facial asymmetry. The jaw and zygomatic arch were deviated to the left side. The jaw was slightly open. The skin around the eyes and nares was mildly encrousted. The globe position seemed normal (Figures 1 and 2). The left globe seemed to be slightly more enophthalmic, and there was more third eyelid protrusion than at the right globe. Both eyes displayed mild blepharospasm. Manipulation of the head was painful.

Vision tests including menace responses were negative. Pupillary light reflexes and dazzle reflexes were negative in both eyes. The palpebral reflex was positive in both eyes. For tonometry, the rebound tonometer (TonoVet, ICare Oy, Finland) was used. The intraocular pressure (IOP) of the left eye was 14 mmHg and of the right eye was 10 mmHg. Slit lamp biomicroscopy (Keeler PSL Classic, Windsor, Berkshire, United Kingdom) revealed bilateral mild conjunctival chemosis. The cornea and visible parts of the sclera appeared to be within normal limits.

The right anterior chamber was extremely shallow due to an anterior displacement of the iris. The iris was bulged anteriorly, adjacent to the corneal endothelium in the temporal part, and appeared stretched. Hyphaema, fibrin and marked aqueous humour flare were present medially. The pupil was miotic, fixed and displaced medially. The right lens and the ocular fundus could not be visualised.

The left anterior chamber was shallow, and flare, fibrin and hyphaema were present. The pupil was miotic and fixed, and the iris was also displaced anteriorly. The left lens and the ocular fundus could not be visualised.

As the posterior segment could not be visualised, further diagnostic evaluation was required. Ultrasound and CT were performed.

### 2.4. Imaging Findings

**Figure 3:**
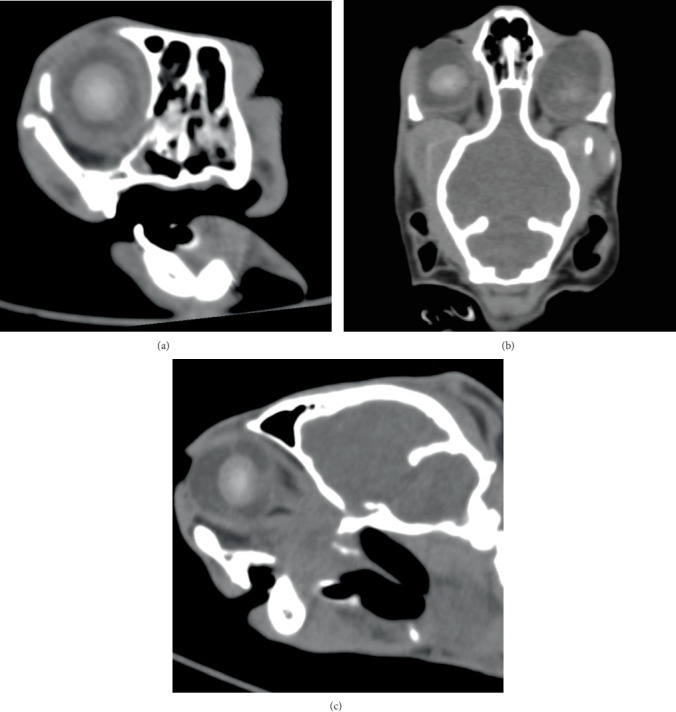
CT of the right ocular globe (medium frequency kernel), right side of the patient is on the left and oblique multiplanar reconstruction—(a) transverse, (b) dorsal and (c) sagittal—centred on the right globe.

**Figure 4:**
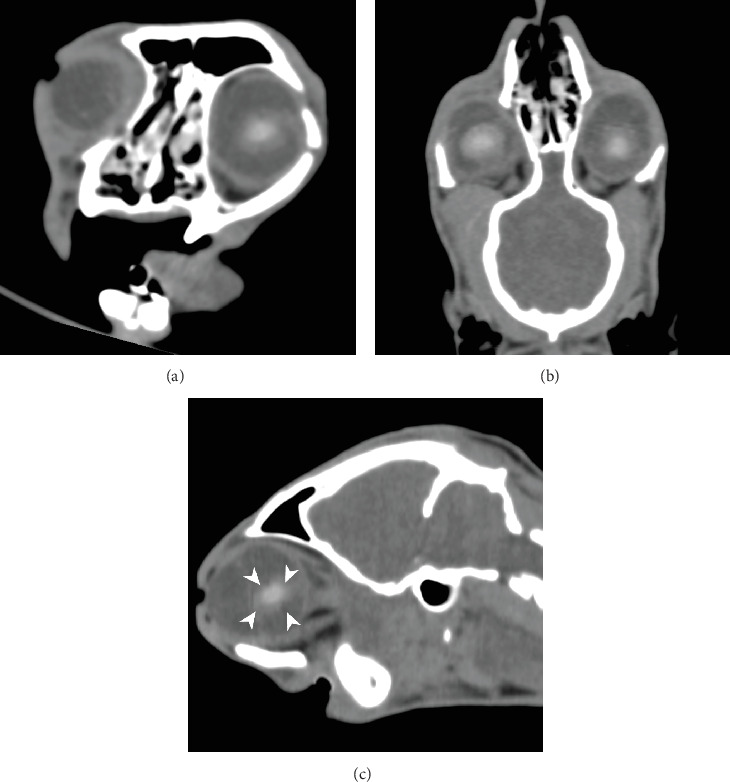
CT of the left ocular globe (medium frequency kernel), right side of the patient is on the left and oblique multiplanar reconstruction—(a) transverse, (b) dorsal and (c) sagittal—centred on the left globe. Note the dislocated ill-defined appearance of the lens (white arrowheads).

The cat was anaesthetised for diagnostic imaging using diazepam (0.3 mg/kg IV, ZIAPAM, 5 mg/mL, O'ZOO GmbH, Ludwigslust, Germany), ketamine (2 mg/kg IV, Anesketin, Eurovet Animal Health B.V., Bladel, the Netherlands) and propofol (Narcofol, CP-Pharma Handelsgesellschaft mbH, Burgdorf, Germany) IV to effect. During CT, anaesthesia was maintained using isoflurane (Isofluran CP 1 mL/mL, CP-Pharma Handelsgesellschaft mbH, Burgdorf, Germany). CT (Somatom Definition AS, Siemens AG, Erlangen, Germany) of the head was performed in sternal recumbency with 120 kV, 300 mAs and slice thickness of 0.75 mm without contrast for assessment of facial fractures, including the temporomandibular joint injury and the eye. Several fractures were identified: fracture of the right mandibular ramus, fracture of the left mandibular condyle, mandibular symphyseal separation with cerclage wire, multiple bilateral maxillary fractures, fractures of the hard palate, bilateral fracture of the zygomatic arch and bilateral fractured pterygoid process. There was soft tissue attenuating material bilateral in the nasal cavity, compatible with (coagulated) blood after initial epistaxis. There was no evidence of brain haemorrhage. There was a difference in the rostrocaudal diameter of the right ocular globe (20 mm) compared to the left globe (23 mm). There was bilateral hyperattenuating material within the anterior chamber compatible with hyphaema. The right ocular globe was flattened caudally with a mild concave shape ventrolaterally. The lens was in a normal position with a diameter of 12 mm and a central attenuation of 151 HU. Within the vitreous chamber, heterogeneous streaky hyperattenuating material (40 HU) was evident (Figure 3). The left ocular globe had a mildly ovoid shape. The shape of the lens was lost, irregular and ill-defined. The lens material was displaced caudally into the vitreous chamber with a central attenuation of 139 HU. Measurement of the diameter was impossible, but compared to the right side, the volume of the lens seemed reduced. Caudal to the lens, within the vitreous chamber, heterogeneous streaky hyperattenuating material (40 HU) was evident (Figure 4). Based on CT findings, right ocular globe rupture, left ocular lens luxation and rupture and bilateral hyphaema and vitreal haemorrhage were assumed.

**Figure 5:**
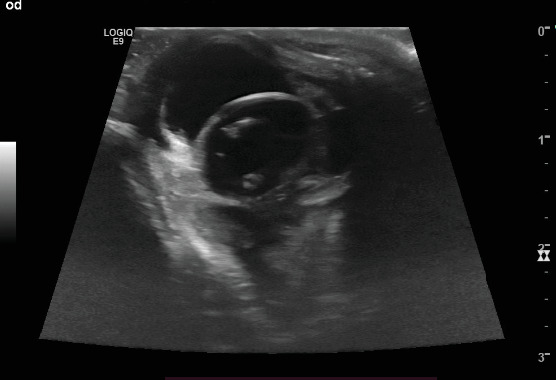
Ultrasound image of the right eye, horizontal transducer position (9 o'clock position); temporal is to the left.

**Figure 6:**
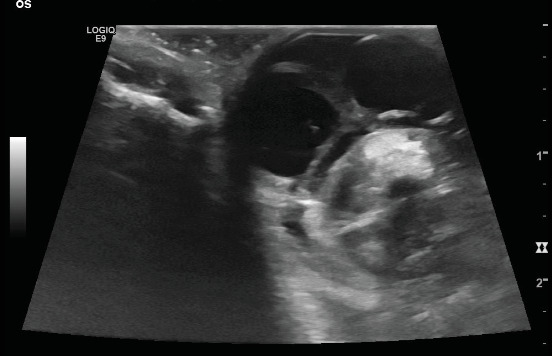
Ultrasound image of the left eye, horizontal transducer position (9 o'clock position); nasal is to the left.

Ultrasound examination of the eyes was performed subsequently to confirm CT findings. Transcorneal ocular ultrasound was performed using topical proparacaine (Proparakain-POS 0.5%, URSAPHARM Arzneimittel GmbH, Saarbrücken, Germany) and an 18 mHz linear probe (L8-L18i-D Hockey Stick Probe, GE Logiq E9, GE Healthcare, Solingen, Germany). The shape of the right globe appeared irregular, and hypoechoic fluid was suspected in the retrobulbar space. The lens was well defined, with two small central hyperechoic spots. Within the vitreous, hyperechoic material was present (Figure 5). The left ocular globe appeared mildly ovoid in shape. There was a thin hyperechoic lamellar structure visible within the anterior chamber, most likely a displaced part of the iris. The shape of the lens was lost; instead, an irregularly shaped, ill-defined, heterogeneous hyperechoic structure was visible within the vitreous. Hyperechoic material was present caudal to the suspected lens within the vitreal chamber (Figure 6).

Based on ultrasound examination, right ocular globe rupture, posterior left ocular lens luxation and rupture and bilateral vitreal haemorrhage were assumed. Additionally, anterior synechia of the iris to the cornea in the right eye and posterior synechia in the left eye were suspected.

Due to the overall severity of injuries and the bilateral loss of vision, the owners elected for the euthanasia of the cat and declined histopathological examination.

## 3. Discussion

To the authors' knowledge, this is the first report describing the CT and ultrasonographic appearance of globe rupture and lens rupture in the veterinary literature.

The findings on CT and ocular ultrasound supporting the diagnosis of globe rupture were consistent with CT features of globe rupture reported in human patients. These include globe deformity or wall irregularity, destruction or dislocation of the lens, intraocular haemorrhage and shallow anterior chamber. In some cases, intraocular foreign bodies and gas can also be found [11]. Intraocular haemorrhage was observed as hyphaema during ophthalmologic examination as well as hyperattenuating material within the vitreal chamber on CT. The material within the vitreal chamber had an attenuation of approximately 40 HU, which is compatible with blood [12].

The ill-defined appearance of the left lens in CT (Figure 3) and ultrasound (Figure 5) is most likely due to rupture of the lens capsule and subsequent leakage of lens material and concurrent fibrinous or inflammatory cellular material, which is suggestive of capsular disruption [7]. The lens appeared smaller and ill-defined than on the contralateral side and also less hyperattenuating. A lower attenuation is reported for the lens in traumatic cataract [13]. This finding is also compatible with the heterogeneous hyperechogenic appearance of the lens in ultrasound. Therefore, we assumed traumatic lens rupture to be likely. To our knowledge, there is no literature available describing CT imaging features of lens rupture in cats. In the normal feline eye, the lens is well-defined with a diameter of approximately 12.5 mm and an attenuation of approximately 150 HU [14]. The thickness of the normal feline lens is 7.7 ± 0.5 mm [15]. Additionally, the lens was displaced, compatible with rupture of the zonular attachments and luxation of the lens [16].

In this case, the trauma was unknown; the most common trauma causes in cats are car accidents and “high-rise” accidents. Trauma is a known cause for ocular globe rupture, and ocular lens rupture due to blunt trauma is described in human ophthalmologic literature [17, 18]. As the trauma was already 2 weeks prior to presentation, penetrating injury should also be considered but seemed less likely due to the variety of fractures and bilateral eye involvement, rather indicating blunt trauma. In chronic penetrating injuries, the corneal wound can seal, and the anterior chamber can reform by the time of examination [4].

As owners declined postmortem examination, the validity of this case report is limited, and it was not possible to finally confirm globe and lens rupture, but both ophthalmologic examination and imaging findings support the diagnosis.

This case demonstrates the important role of ultrasound and CT to assess orbital trauma in patients with limited visibility of the posterior segment of the eye due to hyphaema.

## Data Availability

All data relevant to the case is included in this article.
